# Auditory hallucinations, top-down processing and language perception: a general population study

**DOI:** 10.1017/S003329171800380X

**Published:** 2019-01-04

**Authors:** J. N. de Boer, M. M. J. Linszen, J. de Vries, M. J. L. Schutte, M. J. H. Begemann, S. M. Heringa, M. M. Bohlken, K. Hugdahl, A. Aleman, F. N. K. Wijnen, I. E. C. Sommer

**Affiliations:** 1 Department of Psychiatry, University Medical Center Utrecht, Utrecht University & Brain Center Rudolf Magnus, Utrecht, The Netherlands; 2 Department of Neuroscience and Department of Psychiatry, University of Groningen, University Medical Center Groningen, Groningen, The Netherlands; 3 Department of Biological and Medical Psychology, University of Bergen, Norway; 4 Division of Psychiatry, Haukeland University Hospital, Bergen, Norway; 5 Utrecht University, Utrecht Institute of Linguistics OTS, Utrecht, The Netherlands

**Keywords:** Auditory perception, bottom-up, hallucinations, language, top-down

## Abstract

**Background:**

Studies investigating the underlying mechanisms of hallucinations in patients with schizophrenia suggest that an imbalance in top-down expectations *v.* bottom-up processing underlies these errors in perception. This study evaluates this hypothesis by testing if individuals drawn from the general population who have had auditory hallucinations (AH) have more misperceptions in auditory language perception than those who have never hallucinated.

**Methods:**

We used an online survey to determine the presence of hallucinations. Participants filled out the Questionnaire for Psychotic Experiences and participated in an auditory verbal recognition task to assess both correct perceptions (hits) and misperceptions (false alarms). A hearing test was performed to screen for hearing problems.

**Results:**

A total of 5115 individuals from the general Dutch population participated in this study. Participants who reported AH in the week preceding the test had a higher false alarm rate in their auditory perception compared with those without such (recent) experiences. The more recent the AH were experienced, the more mistakes participants made. While the presence of verbal AH (AVH) was predictive for false alarm rate in auditory language perception, the presence of non-verbal or visual hallucinations were not.

**Conclusions:**

The presence of AVH predicted false alarm rate in auditory language perception, whereas the presence of non-verbal auditory or visual hallucinations was not, suggesting that enhanced top-down processing does not transfer across modalities. More false alarms were observed in participants who reported more recent AVHs. This is in line with models of enhanced influence of top-down expectations in persons who hallucinate.

## Introduction

Classical sensory processing theories regard the brain as a device that is purely stimulus driven (Gibson, 1950, 1966). The brain responds to stimuli in a so-called bottom-up fashion; each percept is generated anew by recombining features from sensory input (Engel *et al*., 2001). More recent theories suggest that the brain is better understood as an active, adaptive system that engages with input from the sensory systems. A key concept is ‘top-down processing’, which refers to the idea that perception is guided by expectations based on previous experiences. This idea is a cornerstone of recent Bayesian models of perception (Stocker and Simoncelli, 2006*b*). Employing top-down information enables a faster processing of sensory information (Fenske *et al*., 2006; O'Callaghan *et al*., 2017). The degree to which top-down processing is at play is assumed to be variable. When sensory input is degraded, distorted (e.g. due to limited channel capacity of a communication device) or ambiguous, the expectations created through top-down processes weigh heavier in interpreting the sensory input. Hence, current models propose that a dynamic balance between bottom-up and top-down processing is necessary for accurate perception (Stocker and Simoncelli, 2006*a*). This also holds for the perception and interpretation of language: the brain is a ‘prediction machine’ wherein top-down expectations continuously predict bottom-up information (Van Berkum, 2010).

Top-down processes play an important role in speech perception and the perceptual learning involved in comprehending strongly accented or distorted speech and associating highly variable speech sounds with the correct phonemic categories (Davis and Johnsrude, 2007). Thus, cognitive expectations often determine what we hear. These top-down expectations greatly speed up processing and thereby increase communication efficiency, since we already activate the words we expect to hear. Notably, however, a disadvantage of such high-speed processing is that it is more error prone in situations where these expectations are not met.

Several authors have argued that errors in processing sensory information might be fundamental for the development of hallucinations in schizophrenia patients (Aleman *et al*., 2003; Dima *et al*., 2010). When top-down sensory expectations are activated without bottom-up sensory input, they may lead to hallucinations when these expectations are not properly deactivated (Grossberg, 1982, 2000). Additionally, several authors showed that the vividness of the expectations was related to perception in schizophrenia patients with hallucinations, as compared with those without (Böcker *et al*., 2000; Aleman *et al*., 2003; Powers *et al*., 2017). Aleman *et al*. (2003) suggested that top-down processing outweighs bottom-up processing in patients with schizophrenia. Consequently, a specific expectation is highly determinative of a perceptual experience. In a similar vein, Hugdahl (2009) hypothesized that an aberrant bottom-up system produces auditory verbal hallucinations in schizophrenia, while an impairment in top-down executive control leads patients to be overwhelmed by the voices. The set hypotheses, as well as a number of others (Jardri *et al*., 2016; Powers *et al*., 2016; 2017), share the assumption that hallucinations are caused by an imbalance between top-down and bottom-up processing. In support of this assumption, a recent study showed that participants with hallucinations gained more from prior expectations in ambiguous situations (Cassidy *et al*., 2018). A related line of research is that of signal detection, which shows that a balance between attention or focus on a stimulus, the actual sensory input and the cognitive modulation of that input is essential for a correct recognition of perceptual information (Bentall and Slade, 1985; Servan-Schreiber *et al*., 1996; Sarter *et al*., 2005).

While hallucinations are well-known as a core symptom of schizophrenia (Shergill *et al*., 1998), they are also associated with a wide variety of psychological disorders such as mood and anxiety disorders and personality disorders as well as in healthy individuals (Posey and Losch, 1983; Bentall, 1990; Tien, 1991; Honig *et al*., 1998; Johns *et al*., 2004; Graham Scott *et al*., 2007; Sommer *et al*., 2008; Korsnes *et al*., 2010; Wigman *et al*., 2012; Kelleher *et al*., 2014).

Although several studies have established a relation between auditory hallucinations and an increased influence from top-down processing relative to bottom-up processes in schizophrenia patients (Bentall *et al*., 1991; Aleman *et al*., 2003; Hugdahl, 2009, 2015), research into this relation in auditory language perception in the general population is scarce. Some preliminary studies on this topic suggest that non-clinical individuals perform similar to the schizophrenia group on tasks that induce top-down processing (Vercammen and Aleman, 2008; Kompus *et al*., 2013) and on cognitive tasks in general (Waters *et al*., 2012). Studying the influence of top-down processing on hallucinations in a large sample from the general population could provide valuable insights, in particular since it surpasses potential confounding factors such as medication effects or long-term effects of mental diseases. Importantly, it could show that hallucinations in patients may be an abnormal product of an otherwise ‘normal’ neurocognitive mechanism.

Subclinical forms of hallucinations are quite common in the general population, and could inform our understanding of psychotic symptoms in pathology. Similar demographic, genetic, and environmental risk factors observed for psychotic-like experiences and schizophrenia support this hypothesis of a shared pathology (Remberk, 2017). In the current paper, we therefore used the simplest definition of a hallucination, i.e. a perception without an evident source from the environment. These hallucinations are subtle and include other positive disorders of perception (Linszen *et al.*, 2018), and are thought to be a form of an extended psychosis phenotype.

The present study investigates the relationship between the occurrence of auditory hallucinations (AH) and the strength of top-down processes in auditory language perception in the general population. This question was evaluated by means of an experimental design that induces top-down processing, which enabled us to test whether these top-down expectations ‘over-rule’ the bottom-up information in participants with AH.

We used an online auditory verbal recognition task. Participants were presented with a series of separate spoken words at a fixed pace. One of these words was the designated target and participants were instructed to respond only to this word. Some words in the stimulus set were similar to the target, either in form and/or meaning. These similarities (‘distractor cues’) were expected to ‘prime’ the target, i.e. create an (implicit) expectation. Expecting to hear a certain target word will activate top-down processes, which could lead to responses on the distractors (i.e. false alarms) if the predictions are strong enough. Increased responses to distractors would thus indicate increased influence of top-down processes. We hypothesized that participants with AH exhibit stronger influence from top-down processes in their perception than non-hallucinating controls, resulting in a higher number of responses on distracting cues, i.e. a higher false alarm rate in the hallucinating group. We controlled for self-reported cannabis use because it is known to influence auditory signal detection and executive functioning in general (Moskowitz and McGlothlin, 1974; Oomen *et al*., 2018).

## Methods

### Participants

The current study is part of a larger project conducted in The Netherlands titled ‘Zie ik spoken?’ (‘*Do I see ghosts?*’). The overall methodology is described in a separate paper (Linszen *et al*., 2018). Participants from the general population could take part in the study through the project's website (https://www.zieikspoken.nl). The study was promoted at several occasions throughout The Netherlands from September 2016 until May 2017, in cooperation with an annual science festival called ‘Weekend of Science’ based on an initiative from the Dutch Ministry of Education, Culture and Science. Study participation was solicited through several Dutch media channels, including television, radio stations, newspapers as well as several science-related festivals. Inclusion criteria for the current study were (1) being a native speaker of Dutch (to avoid differences in perception based on language fluency), and (2) age of 14 and over. The ethical review board of the University Medical Center Utrecht reviewed this study. All participants agreed to the terms and conditions of the study.

### Procedure and measurements

#### Questionnaire for Psychotic Experiences

The Questionnaire for Psychotic Experiences (QPE) is a questionnaire consisting of 50 items designed to quantify range of psychotic experiences, focusing on hallucinations and delusions (Sommer *et al*., 2018). In contrast to clinical psychosis scales (e.g. Overall and Gorham, 1962; Kay *et al*., 1987), this questionnaire not only inquires after problematic psychotic symptoms, but covers the entire spectrum of psychotic experiences, including misinterpretations, visual illusions, incubus and other sleep-related phenomena, sensed presence, passage hallucinations, and visions, etc. (Blom, 2009). Hallucinations were defined as a perception without an evident source from the environment, making the QPE highly suitable to assess the full spectrum of hallucinations and other positive disorders of perception, and psychotic-like experiences of any origin and any duration.

Here we used the online self-survey version of the QPE, in which the hallucination subscales (one for each of four modalities) start with a screening question that evaluates the presence of hallucinations in that modality. Only when hallucinations are reported to occur, additional questions regarding the nature and severity of these hallucinations are asked.

Based on their answers on the QPE, participants were categorized into four different groups per hallucination modality (auditory, visual, olfactory, and tactile), viz., participants who had had: (1) no experience of a hallucination in that modality in their lifetime (‘no hall), (2) at least one hallucination during their lifetime, but not during the past month (‘hall ever’), (3) at least one hallucination in the past month, but not in the past week (‘hall month’), and (4) at least one hallucination in the past week (‘hall week’). AH in the past month or week were further categorized as being either verbal or non-verbal in nature.

#### Auditory verbal recognition task

After filling out the QPE questions, participants were able to participate in the auditory verbal recognition task via the project website. The task was developed in collaboration with the Dutch company Coolminds® and can be accessed here: https://www.youtube.com/watch?v=mircCIDPQAI.

The auditory recognition task began with a written instruction in which participants were instructed to put on their headphones and adjust the volume to a comfortable level. They were then requested to respond as quickly as possible whenever they heard the Dutch word ‘ijsje’ (*ice-cream*), by pressing the computer's space bar (or the screen in case of a mobile device). They were explicitly instructed to not respond to any other word they might hear. After they had read the instruction page, they started the task by pressing the space bar.

During the task the participants heard both target words (‘ijsje’) and several related and unrelated distractors. Related distractors were either phonologically (by sound) and/or semantically (by meaning) related to the target word. All stimuli were presented several times, both in a female and a male voice. The target was presented eight times, each of the distractors was presented four times. To make the task less predictable, stimuli were presented at variable stimulus intervals. Two of the distractors were programmed to overlap partly in time with an ensuing stimulus; this was done to reduce predictability of the stimulus intervals and data regarding these distractors were left out from all analyses. The time between stimuli varied from 0 to 2 s. The auditory verbal recognition task lasted 1 min in total.

Hit rate was calculated as a proportion of correct responses on target words, while the proportion of responses on distractors was defined as the false alarm rate. Following Signal Detection Theory, the detection sensitivity (or discrimination ability) can be expressed by calculating the sensitivity index (*d*’) (Macmillan and Creelman, 2004).

#### Stimuli selection and recording

Data from the Dutch lexicon project (Brysbaert *et al*., 2015) were used to select stimuli for the auditory verbal recognition task; see Table 1. The following factors were controlled, as they influence the speed of word recognition: average accuracy and response time in a lexicon decision task, word frequency, word length and the number of orthographic neighbors (Yarkoni *et al*., 2008). For additional information on the stimuli, see online Supplementary Methods.

**Table 1.** Linguistic characteristics of the stimuli

Stimulus translation	IPA	Stimulus type	Related to target?	PoS	No. syllables	No. phonemes	RT (ms)	Accur.%	Freq.^a^	Old20^b^
ijsje *ice-cream*	(εisjə)	Target	N/A	Dim., noun	2	4	473	99.5	2.59	1.85
ijs *ice*	(εis)	Distr.	+	Noun	1	2	530	99.9	3.18	1.25
meisje *little girl*	(mεisjə)	Distr.	+	Dim., noun	2	5	492	99.7	2.74	1.75
reisje^c^ *short trip*	(rεisjə)	Distr.	+	Dim., noun	2	5	504	95.0	3.17	1.00
kans *chance*	(kɑns)	Distr.	−	Noun	1	4	496	99.6	2.62	1.05
gebied *area*	(ɣəbi:t)	Distr.	−	Noun	2	5	504	99.8	2.85	1.70
blaadje *little leaf*	(bla:tjə)	Distr.	−	Dim.	2	6	530	99.2	2.40	1.85
Mean ± s.d.					1.7 ± 0.49	4.4 ± 1.27	504.1 ± 20.50	98.96 ± 1.760	2.793 ± 0.2954	1.493 ± 0.3791

N/A, not applicable; IPA, Internal Phonetics Association; Distr., distractor; semant., semantic; phon., phonological; PoS, part of speech; dim., diminutive; No., number; RT, response time; ms, milliseconds; accur., accuracy; freq., word frequencies; Old20, orthographic Levenshtein distance.

a Word frequencies are expressed in Zipf's values; values from 1 to 3 represent low-frequency words and values from 4 to 7 high-frequency words.

b The orthographic Levenshtein distance OLD20 was used to express phonological similarity between the target and the various distractors.

c Data for this stimulus were not available in the Dutch lexicon project, therefore data for the word ‘reis’ (*trip*) are presented here.

#### Hearing test

A free online hearing test developed and validated by the Dutch hearing foundation was used to screen for hearing problems (Leensen *et al*., 2011). Based on age-corrected normative hearing data from the Dutch hearing foundation, participants were characterized as having either normal or reduced hearing. Since not all participants took the hearing test, results of the hearing test were not used for the main analyses to avoid selection bias. Instead, sub-analyses were performed using data from participants who took part in the hearing test.

### Data processing

**Fig. 1.**
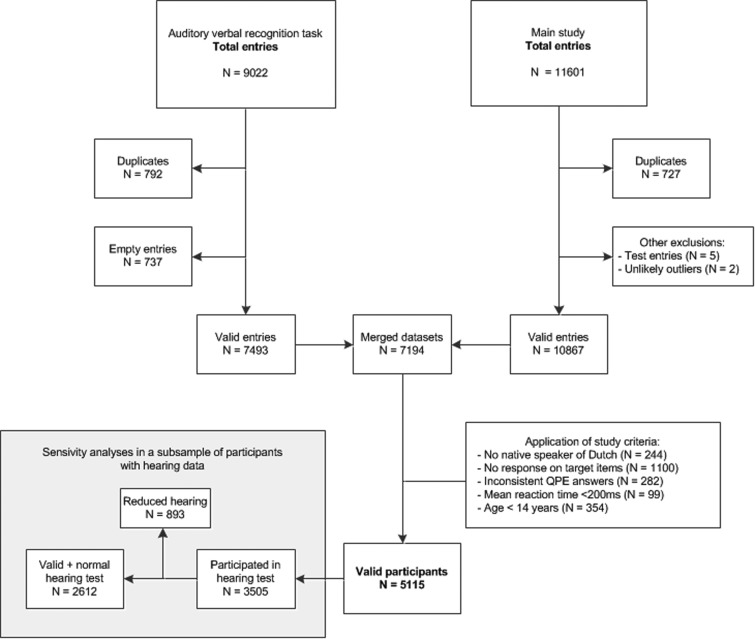
Database flowchart. *n* = number of entries/participants. QPE = Questionnaire for Psychotic Experiences, ms = milliseconds.

A total of 9022 entries were made into the auditory verbal recognition task database (see Fig. 1 for a flowchart). Data from the auditory verbal recognition task were preprocessed to improve validity of the data, for information regarding preprocessing see online Supplementary Methods.

After merging of databases, we then applied the in- and exclusion criteria specific to this study. A total of 5115 valid participants were included in the auditory verbal recognition task. Participants could choose to take a hearing test after completion of the other tasks. Of the 5115 valid participants, 3505 completed the hearing test.

### Statistical analyses

Analyses were performed using IBM SPSS Statistics version 22.0. Participant characteristics were compared between groups using a χ^2^ test for categorical values and an analysis of variance for continuous variables. A Kruskal–Wallis test was used in case an assumption was violated. Relevant test assumptions were assessed visually by evaluating Q–Q plots of the residuals and scatter plots of the predicted values and the unstandardized residuals. A general linear model (GLM) was used to assess the performance of the different hallucinating groups [namely (1) no experience of a hallucination in that modality in their lifetime, (2) at least one hallucination during their lifetime, though not during the past month, (3) at least one hallucination in the past month, but not in the past week, and (4) at least one hallucination in the past week] on the auditory verbal recognition task. A Jonckheere–Terpstra test was used to assess a trend in responses on the auditory recognition in the four AH groups.

## Results

### Questionnaire for Psychotic Experiences

A total of 5115 participants were included in the analyses of the auditory verbal recognition task. More than half (53.6%) of the participants reported the experience of an AH in their lifetime, 25.6% of these participants experienced AH in the past month and 22.7% in the past week. For characteristics of the participants, see Table 2.

**Table 2.** Demographic characteristics

Group	No AH (*n* = 2372)	AH ever (*n* = 1419)	AH month (*n* = 702)	AH week (*n* = 622)	*p* value
Age, mean ± s.d. (IQR)	38.8 ± 15.36 (25–50)	36.1 ± 14.68 (24–47)	33.2 ± 14.20 (22–43)	33.44 ± 14.3 (21–45)	<0.0001
Gender, % female	60.5%	68.6%	74.4%	74.3	<0.0001
Years of education, mean ± s.d. (IQR)	14.4 ± 1.96 (14–16)	14.1 ± 2.07 (14–16)	13.8 ± 2.23 (14–15)	13.6 ± 2.35 (12–15)	<0.0001
Handedness, % left:right:both	11.6:84.9:3.5	12.1:83.5:4.5	12.3:84.3:3.4	10.5:84.4:5.1	0.439
Cannabis use, % yes					<0.0001
No cannabis	59.5%	48.6%	49.7%	49.8%	
Cannabis ever	33.3%	40.3%	36.6%	37.9%	
Cannabis past month	2.2%	3.5%	5.1%	3.4%	
Cannabis past week	5.0%	7.7%	8.5%	8.8%	
Verbal AH	N/A	N/A	51.7%	53.9%	0.434
Visual hallucinations (VH)					<0.0001
No VH	71.2%	50.8%	38.6%	34.1%	
VH ever	17.5%	33.1%	28.5%	20.9%	
VH month	5.0%	7.1%	15.2%	18.2%	
VH week	6.3%	8.9%	17.7%	26.8%	
Tactile hallucinations (TH)					<0.0001
No TH	75.5%	56.6%	45.0%	38.9%	
TH ever	14.8%	27.5%	27.5%	22.3%	
TH month	4.4%	8.2%	12.3%	15.6%	
TH week	5.4%	7.8%	15.2%	23.2%	
Olfactory hallucinations (OH)					<0.0001
No OH	73.7%	59.1%	55.7%	48.9%	
OH ever	15.5%	26.9%	19.4%	15.4%	
OH month	6.0%	7.1%	13.0%	14.3%	
OH week	4.8%	6.9%	12.0%	21.4%	

*n*, sample size; AH, auditory hallucinations; N/A, not applicable; s.d., standard deviation; IQR, interquartile range; No AH, no auditory hallucinations in their lifetime; AH ever, at least one auditory hallucination during their lifetime, though not during the past month; AH month, at least one auditory hallucination in the past month, but not in the past week; AH week, at least one auditory hallucination in the past week.

These four AH groups differed with regard to age, gender, years of education, and the presence of visual, tactile, and olfactory hallucinations. Post-hoc tests revealed that participants with recent hallucinations (week/month) were younger, more often female, and received less education as compared with participants with lifetime AH or no AH in their lifetime (all *p* ⩽ 0.0001). The AH groups also differed in their cannabis use (*p* < 0.0001), participants with lifetime or recent AH overall reported higher cannabis usage.

### Auditory verbal recognition task

**Fig. 2.**
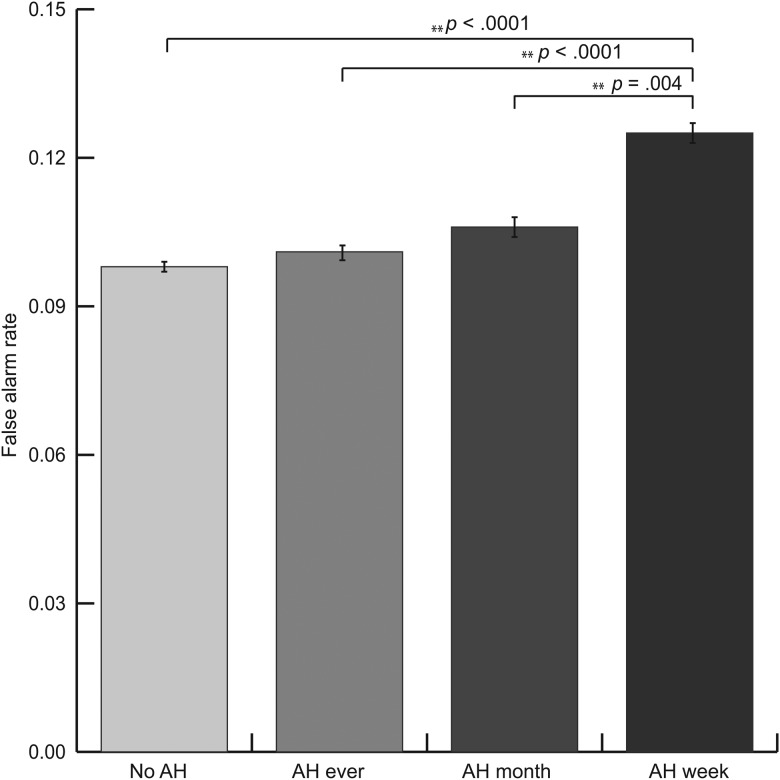
False alarm rate per hallucination group. AH = auditory hallucinations. Error bars indicate standard errors. **Significant at the level of *α* = 0.01. Covariates appearing in the model: age and years of education.

Sensitivity indices were calculated for the four different AH groups, the resulting *d*’ scores were as follows: no AH = 3.688, AH ever = 3.663, AH month = 3.451, AH week = 3.122, showing a clear downward trend in detection sensitivity in relation to increasing recency of AH experiences. Correlation analyses showed that age was negatively correlated with both false alarm rate (*r* = −0.047, *p* = 0.001) and hit rate (*r* = −0.091, *p* < 0.0001). Years of education showed a negative relation with false alarm rate (*r* = −0.114, *p* < 0.0001) and a positive relation with hit rate (*r* = 0.076, *p* < 0.0001). To further explore this effect, a GLM was used to investigate the effect of auditory and visual hallucinations, age, years of education, and gender on the number of correct responses to targets (hit rate) and the number of responses to distractors (false alarm rate). The GLM returned a significant main effect of AH on false alarm rate, *F*_(3,5081)_ = 9.878, *p* *<* 0.0001, partial *η*^2^ = 0.008, but not on the hit rate [*F*_(3,5081)_ = 1.304, *p* = 0.271, partial *η*^2^ = 0.001]. Between-group post-hoc analyses revealed that participants with AH in the past week had more false alarms compared with participants with AH in the past month (*p* = 0.003), lifetime AH (*p* *<* 0.0001), and with no experience of AH in their lifetime (*p* *<* 0.0001), see Fig. 2. The Jonckheere–Terpstra test revealed a significant trend in the false alarm rate over the hallucination groups (*p* < 0.0001) ordered in time from never to the past week.

Age and education had a significant positive effect on both the hit rate [*F*_(1,5081)_ = 47.087, *p* *<* 0.0001 and *F*_(1,5081)_ = 27.254, *p* *<* 0.0001] and a negative effect on false alarm rate [*F*_(1,5081)_ = 5.728, *p* = 0.017 and *F*_(1,5081)_ = 49.463, *p* *<* 0.0001]. The presence of visual hallucinations showed no main effect on auditory recognition (*p* = 0.119). No interaction effects were found between the independent variables.

**Fig. 3.**
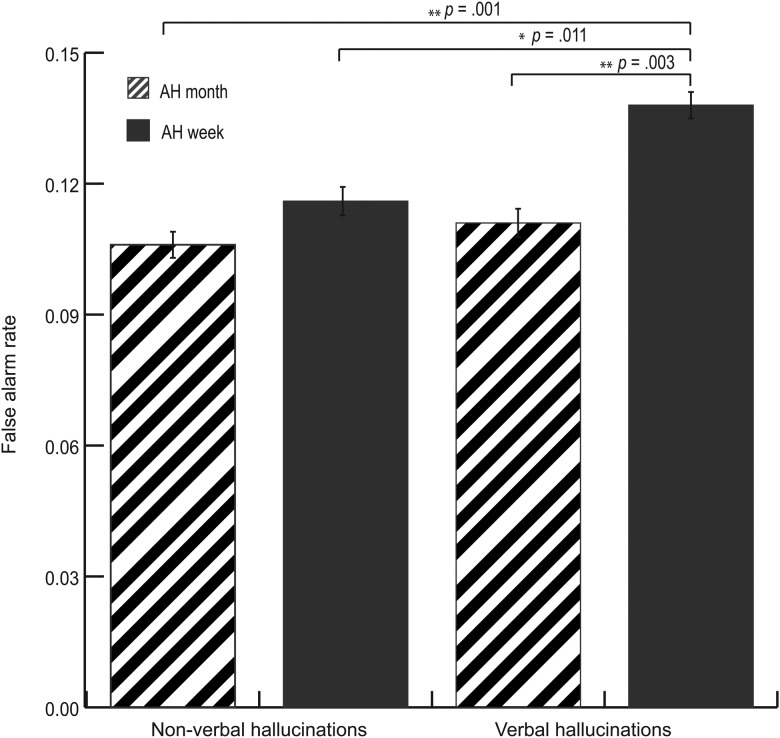
False alarm rate in participants with verbal *v.* non-verbal hallucinations. AH = auditory hallucinations. Error bars indicate standard errors.*Significant at the level of *α* = 0.05, **significant at the level of *α* = 0.01. Covariates appearing in the model: age and years of education. N.B. A higher proportion of distractor signifies more mistakes in auditory perception.

To assess the effect of type of AH, additional analyses were performed for all participants who reported recent AH (i.e. past month or week). For these analyses, AH had been classified as either verbal or non-verbal in nature. The verbal or non-verbal nature of the hallucinations was added to a multivariate GLM, which returned a main positive effect for the presence of verbal hallucinations on the false alarm rate [*F*_(1,1314)_ = 5.601, *p* = 0.001]. Again, no effect was found on the HR. Post-hoc analyses revealed that participants with verbal hallucinations performed worse on the auditory verbal recognition task than participants with non-verbal hallucinations, see Fig. 3.

To assess the influence of cannabis use on task performance, self-reported use was added to a GLM on false alarm rate, which revealed no main effect of cannabis (*p* = 0.627) on task performance, while the effect of the AH remained significant (*p* < 0.0001).

### Effects of hearing acuity

A hearing test was performed to assess hearing difficulties (*n* = 3505); 2612 participants were found to have normal hearing. Participants with deviant hearing were more often men (*p* = 0.045), were older (mean age 45.5 *v.* 34.5) and received fewer years of education as opposed to participants with normal hearing (both *p* *<* 0.0001). Therefore, age, gender, and education were entered as confounders into the analyses. Hearing did not influence the number of responses on distractor stimuli [*F*_(1,3499)_ = 0.612, *p* = 0.434]; however, participants with normal hearing responded to a higher number of target stimuli than participants with deviant hearing test scores [*F*_(1,3499)_ = 40.596, *p* < 0.0001]. Though age had a significant individual effect on the number of responses on target items (*p* < 0.0001), there was no significant interaction between age and hearing problems (*p* = 0.255). Since hearing affects at least part of the performance on the auditory verbal recognition task, all main analyses were replicated in the subgroup of participants with normal hearing (*N* = 2612; mean age 34.5, mean years of education 14.4, and 67% female). These analyses yielded a similar effect on the presence of AH as observed in the main sample (see online Supplementary Table S1), assuring that hearing dysfunction did not influence the results.

## Discussion

We set out to investigate the effects of hallucinations on top-down processing in a large-scale online experiment. Our results indicate that individuals with AH are less sensitive (i.e. reduced discriminatory ability) in their auditory word recognition, as expressed by a higher false alarm rate compared with individuals without AH. This effect increases with the recency of the experienced hallucinations; i.e. if participants experienced the hallucinations more recently, they had more false alarms in the auditory perception task. No relation was found between the experience of hallucinations and the number of target words that were correctly detected. These results are in agreement with models assuming that hallucinations are related to an increased influence of top-down processes as compared with bottom-up processes (Aleman *et al*., 2003; Dima *et al*., 2010). In the present study, the target word was presented before the start of the experiment, which induces an expectation. Our results show that individuals with recent AH are more likely to perceive one of the distractor words as being the target word. In the recent hallucination groups, top-down expectations thus ‘over-ruled’ the actual bottom-up information as presented in the stimuli. These effects were shown to be independent of use of cannabis.

Our results concur with other previous studies. People with increased liability for psychosis reported more speech illusions in random noise, i.e. when no speech was actually presented (Bentall and Slade, 1985; Catalan *et al*., 2014). Furthermore, an increased effect was found of verbal imagery or expectations on subsequent false positives regarding speech in white noise in hallucination-prone individuals (Vercammen and Aleman, 2008; Moseley *et al*., 2016). Our results corroborate and extend previous findings by using a novel task that primes participants for a particular word, and the unusually large sample of participants we were able to test. In addition, we were able to compare verbal *v.* non-verbal AH. In sub-analyses, we found that participants with *verbal* AH in the past week had more false alarm responses than participants with *non-verbal* AH in the past week, showing that the verbal nature of the AH had an independent effect added to the main effect of AH in general. Furthermore, the presence of recent visual hallucination did not predict responses on the auditory verbal recognition task. Our results therefore are in line with the assumption that processes involved in auditory perception are modality-specific.

Interestingly, no relation was found between the frequency of correct hits on target words and the presence of hallucinations. Theoretically, increased expectations of the target word might lead to more responses on the target word as well. This is in line with Signal Detection Theory in which a person's detection sensitivity can be expressed as a standardized measure of the hit rate minus the false alarm rate (Stanislaw and Todorov, 1999; Macmillan, 2002). Our results indicate that the presence of AH decreases a person's sensitivity, but only through their false alarms. The fact that we did not find a difference in both hits and false alarms in our dataset could be due to a plateau effect, as mean response rates on the target words were quite high overall (77%, interquartile range 66.67–1.00%). Furthermore, subtle differences might be less detectable in the target words because the absolute number of times participants heard a target word was much smaller than the number of distractors (8 *v.* 24).

Given the cross-sectional nature of this study, a causal relation between the mistakes in auditory perception and the presence of AH cannot be established. However, our results indicate that the more recent the hallucinations are, the higher the influence of top-down expectations is (more false alarms) which does hint at a more specific effect.

A major strength of this study is its large sample size, which makes it possible to pick up subtle effects and makes the results generalizable to other populations. Furthermore, we were able to provide a mechanism behind the perception of AH in the general populations, proving participants with recent AH make more auditory perception errors.

Though our results were statistically significant, the differences between the four hallucination groups were quite small overall and effect sizes are small. The false alarm rate was only a few percent points higher in participants who experienced AH in the past week compared with the non-hallucinating group. Importantly, however, these effects were found in a non-clinical sample taken from the general population. Our results indicate that even a once in a lifetime experience of an auditory hallucination is associated with aberrant auditory language perception suggesting a close relationship between both processes that can be further studied in general population samples.

An interesting finding in our study is that the influence of top-down predictions appears to be domain-specific, since the presence of recent auditory *verbal* hallucinations was predictive for perception errors, while visual and non-verbal hallucinations were not. Previous studies have shown that imbalanced top-down processes are domain-specific, showing that patients with AH have difficulties with auditory perception but not with visual perception (Böcker *et al*., 2000; Aleman *et al*., 2002). This relates to the broader question whether language can be characterized as domain-specific or a more general cognitive process, which remains a topic of debate (Blank and Fedorenko, 2017; Frost *et al*., 2017). Domains in this context refer to a range of stimuli that share structural or physical properties such as spoken words, musical tones, or tactile sensations. The question of domain specificity has been addressed in many studies, including processes involved in learning a language (Hauser *et al*., 2002), statistical learning (Frost *et al*., 2015), and memory (Dehaene and Cohen, 2011). Most researchers agree that the language faculty is in part a unique modality; however, it also relies on more general cognitive functions. Our findings also suggest that increased top-down verbal processing (reflected by AVH), but not hallucinating non-verbally, is predictive of word detection performance. It thus appears that specifically top-down processes involved in language processing are affected in subjects who experience AVH, while other top-down processes may remain (relatively) untouched.

Online surveys are a research method that is rapidly gaining popularity in psychiatry. They have specific advantages and disadvantages that make them highly suitable to investigate common phenomena and their risk factors in large samples (Evans and Mathur, 2005; Wright, 2005). Because of their high power, more subtle associations can also become evident. Another advantage is the relative anonymity of participation, which can overcome stigma in studying topics associated with a sense of shame or denial. Our data appear to corroborate this advantage. Over 50% of the participants reported hallucinations, while previous telephone and interview settings found a prevalence of 30–40% (Ohayon, 2000; Johns and Van Os, 2001; Johns *et al*., 2002). The higher percentage in the present study could also be related to the fact that online participation is a choice that is made on individual interest and preferences. In general, more youngsters and more females participate in online surveys, leading to skewed population samples; therefore, we controlled for these factors in our analyses. Furthermore, people affected by the phenomenon under study may be more inclined to participate than those without, which could lead to inflated prevalence estimates. A limitation of our design was the short task duration, which prevented comparison of different trials. A benefit of short tasks however is that the risk of mental exhaustion is limited, which is relevant since both memory and attention span are known to be decreased in schizophrenia patients (Rund, 1998), which may apply to non-clinical participants with hallucinations as well. A final limitation of our design was that we were unable to analyze exact response times; therefore, the temporal aspect of these processes could not be assessed. There were many unknown factors that influenced response time such as Internet connection speed and processing speed of the device, which is also a known pitfall of online surveys.

We show that people with AH make more expectation-based mistakes in their auditory verbal perception, compared with participants without (recent) hallucinations. By contrast, visual hallucinations do not influence auditory perception. Furthermore, we demonstrated a clear trend that the more recent the hallucinations were experienced, the higher number of mistakes was made. More specifically, the presence of auditory *verbal* hallucinations was related to auditory speech perception errors, further indicting a modality-specific effect. Our results support the Bayesian proposition that the larger influence of top-down processes leads to an increased amount of false-positive mistakes in auditory verbal perception, which facilitates AH.

## Data Availability

The data that support the findings of this study are available from the corresponding authors on reasonable request.

## Supplementary material

For supplementary material accompanying this paper visit https://doi.org/10.1017/S003329171800380X. click here to view supplementary material
