# Supplementary material for: Auditory hallucinations, top-down processing and language perception: a general population study
Source: Psychol Med. 2019 Jan 4;49(16):2772–80. doi: 10.1017/S003329171800380X (PMC6877468; doi:10.1017/S003329171800380X)
Supplement: Supplementary file 1 [file S003329171800380Xsup001.docx]

**Supplementary Material**

**Supplementary Methods**

***Auditory stimuli***

A t-test was performed to compare characteristics of the words (such as accuracy and response time), no significant differences between the characteristics of the target and the distractors were found (all p>.10). This indicates that auditory recognition of these words will not be based on intrinsic properties of the different words. Two Dutch speaking volunteers who were unaware of the aims of the study, were asked to read all words aloud twice in a normal speaking voice. The order of the words was randomized during the recording phase, to counteract potential word order effects in stress patterns. The stimuli were recorded onto a TASCAM DR-40 solid state recorder using an AKG-C544l head-worn cardioid microphone. Speech was digitally recorded in a single channel (mono) at a sampling rate of 44,100 kHz with 16-bit quantization.

***Data processing***

First, all identical duplicates were removed. In case of repeated entries for a single participant, the first non-empty entry was used for analysis to avoid learning effects in the later entries. All entries in which no response was registered were removed; these were due to incompatibility issues with iOS devices.

Validation of the data was further improved by removing data that looked suspect. This was required because participants were able to access the experiment from their mobile device anywhere they wanted without supervision. Therefore, we removed participants that failed to respond to any target stimulus (n=1100; mean age=38.5, mean years of education=14.0). Furthermore participants with an overall mean response time of <200ms were removed from further analyses (n=99). This cutoff was based on eye tracking studies that show that the earliest stages of spoken language comprehension start at 200ms (Allopenna *et al.* 1998).

**Supplementary Table**

**Table S1. GLM in participants with normal hearing**

|  | Pillai’s Trace | F | *p-*value |
| --- | --- | --- | --- |
| Age | .004 | 5.746 | .003** |
| Hit rate | - | 11.376 | .004** |
| False-alarm rate | - | .014 | <.0001** |
| Years of education | .016 | 20.901 | <.0001** |
| Hit rate | - | 2.845 | .092 |
| False-alarm rate | - | 35.432 | <.0001** |
| Gender | .001 | 1.070 | .343 |
| Hit rate | - | - | - |
| False-alarm rate | - | - | - |
| Auditory Hallucinations | .006 | 2.418 | .025* |
| Hit rate | - | 1.560 | .197 |
| False-alarm rate | - | 3.686 | .012* |

*Table legend: * = significant at the α = .05 level ** = significant at the α = .01 level.*
